# Hydroxychloroquine-Chloroquine, QT-Prolongation, and Major Adverse Cardiac Events: A Meta-analysis and Scoping Review

**DOI:** 10.1177/10600280231204969

**Published:** 2023-10-26

**Authors:** Michael Cristian Garcia, Kai La Tsang, Simran Lohit, Jiawen Deng, Tyler Schneider, Jessyca Matos Silva, Lawrence Mbuagbaw, Anne Holbrook

**Affiliations:** 1 Clinical Pharmacology & Toxicology Research Group, St. Joseph’s Healthcare, Hamilton, ON, Canada; 2 Temerty Faculty of Medicine, University of Toronto, Toronto, ON, Canada; 3 Department of Health Research Methods, Evidence, and Impact, McMaster University, Hamilton, ON, Canada; 4 Biostatistics Unit, Father Sean O’Sullivan Research Centre, St. Joseph’s Healthcare, Hamilton, ON, Canada; 5 Division of Clinical Pharmacology & Toxicology, Department of Medicine, St. Joseph’s Healthcare, Hamilton, ON, Canada

**Keywords:** hydroxychloroquine, chloroquine, QT interval, torsades de pointes

## Abstract

**Objectives:**

We aimed to evaluate the high-quality literature on the frequency and nature of major adverse cardiac events (MACE) associated with either hydroxychloroquine (HCQ) or chloroquine (CQ).

**Data sources:**

We searched Medline, Embase, International Pharmaceutical Abstracts, and Cochrane Central from 1996 onward using search strategies created in collaboration with medical science librarians.

**Study selection and data extraction:**

Randomized controlled trials (RCTs) published in English language from January 1996 to September 2022, involving adult patients at least 18 years of age, were selected. Outcomes of interest were death, arrhythmias, syncope, and seizures. Random-effects meta-analyses were performed with a Treatment Arm Continuity Correction for single and double zero event studies.

**Data synthesis:**

By study drug, there were 31 HCQ RCTs (n = 6677), 9 CQ RCTs (n = 622), and 1 combined HCQ-CQ trial (n = 105). Mortality was the most commonly reported MACE at 220 of 255 events (86.3%), with no reports of torsades de pointes or sudden cardiac death. There was no increased risk of MACE with exposure to HCQ-CQ compared with control (risk ratio [RR] = 0.90, 95% CI = 0.69-1.17, *I*^2^ = 0%).

**Relevance to patient care and clinical practice:**

These findings have important implications with respect to patient reassurance and updated guidance for prescribing practices of these medications.

**Conclusions:**

Despite listing as QT-prolonging meds, HCQ-CQ did not increase the risk of MACE.

## Introduction

### Background

The antimalarials chloroquine (CQ) and its derivative hydroxychloroquine (HCQ) were originally indicated to prevent and cure malaria, and since the 1940s have been repurposed to treat rheumatological, immunological, and infectious diseases.^1-3^ Hydroxychloroquine is thought to have fewer safety concerns such as retinopathy, cardiomyopathy, and agranulocytosis.^4-8^ These drugs were used early in the COVID-19 pandemic as potential therapies given their antiviral properties; however, HCQ-CQ proved ineffective for improving COVID-19 mortality, viral clearance, or clinical recovery.^9-13^ Both drugs are classified by CredibleMeds as “known” causes of QT-prolongation (QTP) and torsades de pointes, but the link to clinical outcomes is based mainly on very low-quality evidence including pharmacologic mechanisms and case reports.^14-16^

QT-prolongation is a commonly used surrogate marker for the risk of torsades de pointes and other arrythmias. The Food and Drug Administration (FDA) lists major adverse cardiac events (MACE) related to QTP as torsades de pointes, sudden death, ventricular tachycardia, ventricular fibrillation and flutter, syncope, and seizures.^17^ The data on MACE related to HCQ-CQ prescribed for patients arise mainly from observational studies, pharmacovigilance, preclinical, and pharmacokinetic/pharmacodynamic modeling studies, which are susceptible to biases and confounding. In a 2016 review of the cardiotoxicity of antimalarials in malaria patients, the World Health Organization found no significant difference in risks of cardiotoxicity following exposure to CQ at recommended doses.^18^ A 2021 meta-analysis of 25 observational trials (N = 41 339) and 11 randomized trials (N = 8709) comparing HCQ with active drug, placebo, or observation comparators in COVID-19 patients found no association with mortality when pooling the randomized controlled trials (RCTs) but a 20% lower mortality risk for patients taking HCQ when pooling cohort studies.^19^ Similarly, a large retrospective study of 2.2 million patients with rheumatoid arthritis suggested that HCQ was associated with lower rates of MACE (hazard ratio: 0.827, 95% CI = 0.80-0.86).^20^ These findings all include lower quality evidence.

As part of research aimed to clarify the risk of FDA-defined MACE outcomes associated with medications classified as “Known Risk” QT-prolonging medications, we undertook a scoping review of the RCT data for adult patients exposed to HCQ-CQ to estimate the relative rate of MACE—specifically death, torsades de pointes, ventricular tachyarrhythmia, nonfatal cardiac arrest, syncope, and seizures, compared with placebo, standard of care, or observation.

## Methods

We conducted this scoping review with guidance from the Preferred Reporting Items for Systematic Reviews and Meta-analysis (PRISMA) extension for scoping reviews (PRISMA-ScR).^21^ The completed PRISMA-ScR checklist is included as Supplemental Appendix 1. This review was prospectively registered on Open Science Framework (https://doi.org/10.17605/OSF.IO/ZU9F2).

### Study Identification

We searched Medline, Embase, International Pharmaceutical Abstracts (IPA), and Cochrane CENTRAL from early January 1996 until end-October 2020 with an update to end-September 2022 using search strategies developed with advice from research librarians. Complete search strategies are available in supplementary appendices (see Appendix 2). We also completed supplemental searches on Google Scholar using key text words for placebo-controlled trials, HCQ, CQ, safety, and adverse drug reactions.

### Inclusion Criteria

Our inclusion criteria were as follows: (1) parallel-group or crossover design RCTs of any duration; (2) adult patients defined as 18 years or older; (3) at least one intervention arm where the independent effect of HCQ or CQ (either given alone or with the same active agent in both QTPmed and control arm) was measured; and (4) a comparison arm consisting of placebo, active comparator, or no treatment. We did not exclude trials that did not report any MACE (zero-zero event trials).

### Study Selection

Several authors performed title and abstract screening independently and in duplicate in Covidence.^22^ Potentially relevant publications were assessed further during in-duplicate full-text screening. We excluded RCTs involving only pediatric patients or mixed pediatric-adult populations where the adult data could not be separated. Randomized controlled trials involving healthy volunteers, protocols, and conference abstracts were excluded. We did not include any observational studies including health system data base analyses or postmarketing studies due to biases and confounders inherent to these study designs. Studies not written in English language were also excluded. Disagreements were resolved by a third author to attain consensus.

### Extraction of Study Characteristics and Outcomes

Study characteristics and outcomes were extracted independently by pairs of reviewers. We pilot-tested and used a prospectively designed standardized data-extraction form to extract details on study characteristics, dose and frequency of study drugs, participant characteristics, and MACEs. Our primary outcome was the MACE composite adapted from FDA, which included all-cause mortality, sudden cardiac death, nonfatal cardiac arrest, torsades de pointes, ventricular tachycardia, ventricular fibrillation, syncope, and seizure.^17^ We extracted data on whether any of our MACE or QTP were prespecified outcomes of interest, or whether patients with electrocardiogram (ECG) abnormalities or cardiac comorbidities were excluded. Risk-of-bias assessment included allocation concealment, degree of blinding, and loss to follow-up. Discrepancies and disagreements were resolved through discussion or adjudicated by a third senior author.

### Statistical Analysis

We used the *meta 6.0* package in R to conduct the meta-analysis and pool trial data. The unit of analysis was individuals exposed to study medications (HCQ-CQ or control) and data are reported as incident events (number of participants who developed MACE during the study period). Analysis included all participants, including dropouts, to minimize bias due to differences in dropout numbers between groups. To retain studies with zero events in both arms, we used Mantel-Haenzel odds ratios (MH ORs) with Treatment Arm Continuity Correction (TACC) along with 95% CIs.^23^ Publication bias was assessed using funnel plots and Harbord test. As a post hoc analysis, we performed a meta-regression in R of the effect based on the cumulative CQ base dose on MACE, similar to the methodology of other systematic reviews involving CQ compounds.^24,25^

Statistical heterogeneity was assessed using the *I*^2^ statistic, with values of 50% or more indicating a moderate level of heterogeneity.^26^ Statistical significance was set at 2-sided α of 0.05. In trials that had more than 2 intervention groups, for example 2 arms of HCQ or CQ that differed by dose, we preserved randomization but collapsed the multiple intervention arms into single treatment arms.

### Subgroup and Sensitivity Analyses

Prespecified subgroup analyses were conducted for each individual MACE outcome, exclusion of cardiac disease or risk factors or ECG abnormalities, >50% versus < 50% female participants, mean/median age < 65 versus ≥65 years, MACE outcomes or QTP as prespecified outcomes of interest, and HCQ versus CQ alone. Our sensitivity analyses involved investigating the robustness of our effect estimates when using alternative zero-event correction approaches such as the Peto statistical model approach (excluding zero event trials), and the exclusion of all-cause mortality from the overall MACE composite.

## Results

**Figure 1.**
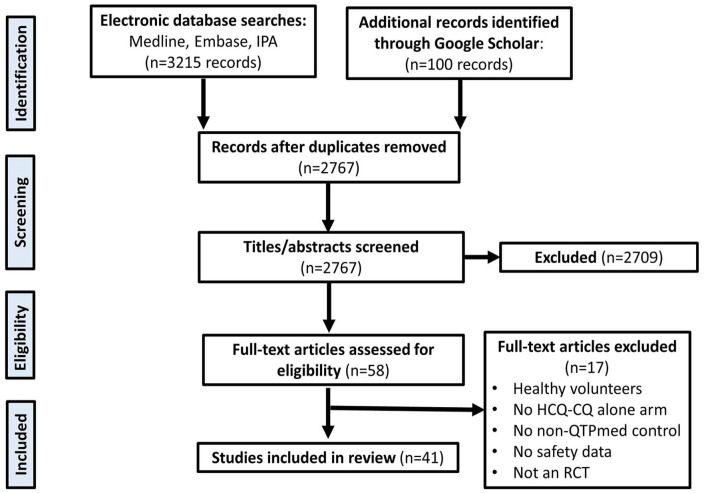
PRISMA flow diagram of the included studies in the meta-analysis. Abbreviations: HCQ-CQ, hydroxychloroquine-chloroquine; IPA, International Pharmaceutical Abstracts; PRISMA, Preferred Reporting Items for Systematic Reviews and Meta-analysis; QTP, QT-prolongation; RCT, randomized controlled trial.

A total of 3215 records were retrieved from our literature searches. After de-duplication, 2767 records remained for evaluation. Title and abstract screening yielded 58 eligible records for full-text evaluation, which resulted in 41 randomized trials included in this scoping review and meta-analysis (N = 7404). See Figure 1 for PRISMA flowchart of the study selection process.

### Study Characteristics

Characteristics of the trials are summarized in Table 1. Of 41 trials, 31 investigated HCQ only,^27-57^ 9 CQ only,^58-66^ and 1 studied HCQ or CQ analyzed as a single arm.^67^ The mean sample size was 197 (SD = 26.7), mean 59.2% (SD = 26.6) female, and most trials (n = 38, 92.3%) reported mean or median ages of their participants to be younger than 65 years. Location of the trials was mainly North America (n = 14, 34.1%), Europe (n = 8, 19.5%), and Asia (n = 8 trials, 19.5%). The mean duration of follow-up was 26.1 weeks (SD = 39.5). Only 2 trials reported data on cardiac comorbidities at baseline.^34,54^ Thirteen trials (31.7%) listed at least one MACE as an outcome of interest in their methods and 8 trials (19.5%) examined QTP during the trial.

**Table 1.** The Main Characteristics of Studies Included in the Meta-analysis.

Study	Geographic region	Duration of follow-up (weeks)	Patient population	Age^a^ Female (F) %	Treatment arms	Sample size	Comparator
Arnaout et al^58^	North America	6	Breast cancer	Age: 56.8 (9.2) F(%): 100	CQ 500 mg OD	70	Placebo
Boulware et al^29^	North America	2	Adults exposed to COVID-19	Age: HCQ: 41 (33-51); Ctrl: 40 (32-50); F(%): 51.6	HCQ 800 mg once; or 600 mg 6-8 hours; or 600 mg OD ×4d	821	Placebo
Cavalcanti et al^30^	South America	2.1	COVID-19 patients	Age: 50.3 (14.6) F(%): 41.7	HCQ 400 mg BID	665	Standard of care (unspecified)
Gottenberg et al^36^	Europe	48	Primary Sjögren Syndrome	Age: HCQ: 55.6 (13.9); Ctrl: 56.3 (11.9) F(%): 91.7	HCQ 400 mg OD	120	Placebo
Jokar et al^37^	Asia	24	Knee osteoarthritis	Age: HCQ: 47.6 (8.54); Ctrl: 48.3 (11.14) F(%): 84.3	HCQ 200 mg BID	51	Placebo
Kedor et al^39^	Europe	56	Inflammatory and erosive hand osteoarthritis	Age: HCQ: 52.4 (8.1); Ctrl: 50.2 (6.6); F(%): 98.1	HCQ 200 mg/d; 200 and 400 mg every other day; or 200 mg BID	156	Placebo
Das et al^33^	Asia	12	Rheumatoid arthritis	Age: HCQ: 40.3 (9.7); Ctrl: 40.0 (10.9) F(%): 78.7	HCQ 200 mg BID	122	Placebo
Liu et al^43^	North America	2	COVID-19 patients	Age range: 22-84 F(%): 63.5	HCQ 200 mg BID	51	Observation (unspecified)
Meinao et al^62^	South America	52	Systemic lupus erythematosus	Mean age: 31.7 F(%): 87.5	CQ 250 mg OD	24	Placebo
Mitja et al^44^	Europe	4	COVID-19 patients	Age: HCQ: 41.7 (12.6); Ctrl: 41.3 (12.4) F(%): 58.6	HCQ 400 mg OD	353	Standard of care (unspecified)
Paton et al^46^	Europe	48	HIV patients	Age: HCQ: 31.7 (7.7); Ctrl: 38.3 (10.8); F(%): 7.2	HCQ 400 mg OD	83	Placebo
Reis et al^47^	South America	12	COVID-19 patients	Age: 53 (18-94) F(%): 55	HCQ 400 mg OD	685	Placebo
Rojas Puentes et al^64^	North America	182	Brain metastases	Age: CQ: 55.7 (13); Ctrl: 52.0 (10.6) F(%): 79	CQ 150 mg OD	78	Placebo
Schwartz et al^48^	North America	4	COVID-19 patients	Age: HCQ: 46.7 (11.5); Ctrl: 46.9 (11.0); F(%): 45.0	HCQ 200 mg BID	124	Placebo
Self et al^49^	North America	4	COVID-19 patients	Age: HCQ: 58 (45-96); Ctrl: 57(43-68); F(%): 44.3	400 mg of HCQ BID ×2 doses, then 200 mg BID	479	Placebo
Solomon et al^50^	North America	16	Rheumatoid arthritis	Age: 56 (11.4) F(%): 95.7	HCQ 6.5 mg/kg/d	23	Placebo
Sotelo et al^65^	North America	86	Glioblastoma multiforme	Age: CQ: 40.8 (11.8); Ctrl: 46.1 (12.7); F(%): 43.3	CQ 150 mg OD	30	Placebo
Tang et al^51^	Asia	4	COVID-19 patients	Age: HCQ: 48.0 (14.1); Ctrl: 44.1 (15.0) F(%): 45.3	HCQ 1200 mg OD ×3d, then 800 mg OD	150	Standard of care (National COVID-19 clinical practice guidelines—China)
Toledo et al^52^	North America	13	Insulin-resistant adults without rheumatic disease	Age: HCQ: 47.9 (14.4); Ctrl: 44.6 (14.7); F(%): 73.5	HCQ 400 mg OD	34	Placebo
Ulander et al^53^	Europe	52	Myocardial infarction patients	Age: HCQ: 59.9 (9.5); Ctrl: 58.5 (9.9); F(%): 20.8	HCQ 300 mg OD	125	Placebo
Ulrich et al^54^	North America	4	COVID-19 patients	Age: HCQ: 66.5 (16.4); Ctrl: 65.8 (16.0); F(%): 40.6	HCQ 400 mg BID ×1d, then 200 mg BID ×2-5 d	128	Placebo
Van Gool et al^55^	Europe	78	Alzheimer disease	Age: HCQ: 70.4 (8.3); Ctrl: 70.7 (8.5); F(%): 57.7	HCQ 400 mg or 200 mg OD	168	Placebo
Yoon et al^57^	Asia	16	Primary Sjögren syndrome patients	Age: HCQ: 59.4 (9.42); Ctrl: 55 (9.72); F(%): NR	HCQ 300 mg OD	26	Placebo
Avezum et al^27^	South America	4.3	COVID-19	Age: 45 (36-56) F(%): 53	HCQ 400 mg BID ×1 d, then 400 mg OD ×7 d	1372	Placebo
Boonpiyathad et al^28^	Asia	12	Chronic spontaneous urticaria	Age: HCQ: 33 (12.11); Ctrl: 33.95 (11.91) F(%): 85.3%	HCQ 400 mg OD	48	Placebo
Tsakonas et al^32^	North America	156	Systemic lupus erythematosus	Age: HCQ: 45 (14); Ctrl: 44 (16) F(%): 97	HCQ: 100-400 mg OD	47	Placebo
Chen et al^31^	Asia	0.9	COVID-19	Age: 44.7 (15.3) F(%): 53.2%	HCQ: 400 mg OD	62	Standard of care (oxygen, antivirals, antibacterial, immunoglobulin +/− corticosteroids)
Dubee et al^34^	Multiple regions	4	COVID-19	Age: HCQ: 76 (60-85); Ctrl: 78 (57-87) F(%): 51.6	HCQ: 200 mg BID	250	Placebo
Gonzalez et al^35^	North America	4.2	COVID-19	Age: 53.8 (16.9) F(%): 37.8	HCQ: 400 mg BID ×1d, then 200 mg BID ×4 days	106	Placebo
Kavanaugh et al^38^	North America	12	Systemic lupus erythematosus	Age: NR F(%): 100	HCQ: 400 mg OD; 800 mg OD	17	Placebo
Kingsbury et al^40^	Europe	52	Hand osteoarthritis	Age: HCQ: 62.8 (9.1); Ctrl: 62.5 (9.2) F(%): 81.5	HCQ: 200, 300, 400 mg OD	248	Placebo
Lee et al^41^	Europe	24	Hand osteoarthritis	Age: HCQ: 58.3 (7); Ctrl: 57.7 (8.2) F(%): 86	HCQ: 400 mg OD	202	Placebo
Levy et al^42^	South America	12	Systemic lupus erythematosus	Age: HCQ: 29 (3); Ctrl: 29 (4) F(%): 100	HCQ: NR	20	Placebo
Omrani et al^45^	Multiple regions	2	COVID-19	Age: HCQ: 40 (31-47); Ctrl: 41 (31-47) F(%): 1.7	HCQ: 600 mg OD	456	Placebo
Rea-Neto et al^67^	South America	4	COVID-19	Age: CQ: 54.7 (12.1); Ctrl: 52.8 (12.6) F(%): 33.4	CQ: 250 mg BID ×1d, then 450 mg OD ×5d; HCQ 400 mg BID ×1d, then 400 mg OD ×5d	142	Standard of care (unspecified)
Yokogawa et al^56^	Asia	16	Cutaneous lupus erythematosus	Age: HCQ: 43.1 (12.8); Ctrl: 41.6 (12.7) F(%): 74	HCQ: 200 mg OD or 400 mg OD	103	Placebo
Baltzan et al^59^	North America	24	Advanced pulmonary sarcoidosis	Age^b^: 42.5 (29-67) F(%): 41.7	CQ: 250 mg OD	24	Observation^b^ systemic corticosteroids
Borges et al^60^	South America	1	Dengue fever	Age: 31.64 (11.74) F(%): 51.3	CQ: 300 mg BID	37	Placebo
De Lamballerie et al^61^	Africa	3.4	Acute chikungunya infection	Age: NR F(%): NR	CQ: 300 mg BID ×3d, then 300 mg OD ×2d	54	Placebo
Peymani et al^63^	Eastern Mediterranean	12	Hepatitis C virus nonresponders	Age^b^: CQ: 50 (45-52); Ctrl: 49 (25-60) F(%): 0	CQ: 150 mg OD	10	Placebo
Tricou et al^66^	Asia	2	Dengue fever	Age: CQ: 22 (18-27); Ctrl: 22 (19-28) F(%): 31.6	CQ: 600 mg ×2d, then 300 mg ×1d	307	Placebo

Abbreviations: BID, twice daily; CQ, chloroquine; Ctrl, control; HCQ, hydroxychloroquine; IQR, interquartile range; NR, not reported; OD, once daily.

a Age reported as median (IQR) or mean (SD) unless otherwise indicated.

b Mean (range).

Indications for the HCQ-CQ varied, the 3 most common being 15 trials for COVID-19 (n = 5844)^27,29-31,34,35,43-45,47-49,51,54,67^; 8 trials (n = 405) for patients with immune-mediated conditions, including systematic lupus erythematosus,^32,38,42,56,62^ primary Sjogren syndrome,^36,57^ and chronic spontaneous urticaria^28^; and 6 trials (n = 802) for patients with rheumatic conditions (rheumatoid arthritis, hand osteoarthritis, and knee osteoarthritis).^33,37,39-41,50^ Of the 41 RCTs, 34 (75.6%) used placebo as the comparator,^27-29,32-42,45-50,52-58,60-66^ 5 used active standard of care medications as comparator,^30,31,44,51,67^ and 2 used nothing.^43,59^

With respect to the selected domains of risk of bias in the included trials, 18 of 41 (43.9%) detailed appropriate allocation concealment procedures, but 22 of 41 (53.7%) failed to provide any details on allocation concealment. Most trials, 34 of 41 (82.9%), were at least double-blinded, and most (31/41, 75.6%) reported ≤15% loss to follow-up.^68^

### Prevalence of MACE Outcomes

A total of 255 MACEs were reported in 14 trials,^27,30,34-36,39,40,49,53-55,64,65,67^ with the remainder reporting no events. Of the reported MACE, most (220/255, 86.3%) were deaths. Thirteen trials named a specific MACE as an outcome of interest in their methods,^27,29,30,34,47-49,51,53,54,64,65,67^ of which 8 of 13 (61.5%) reported MACE outcomes during the trial.^27,30,49,53,54,64,65,67^ In contrast, only 5 trials of the 28 (17.9%) that did not prespecify MACE reported MACE incidents.^35,36,39,40,55^

### Meta-analyses of Incident MACE

**Figure 2.**
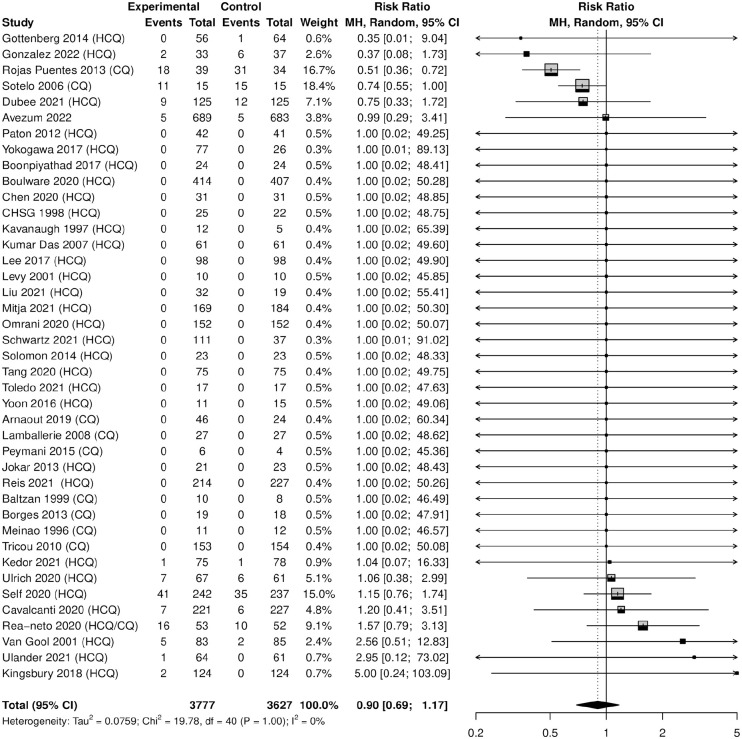
Forest plot of hydroxychloroquine/chloroquine vs control for any MACE. Abbreviations: CQ, chloroquine; HCQ, hydroxychloroquine; MACE, major adverse cardiac events; MH, Mantel-Haenzel.

Hydroxychloroquine-chloroquine was not associated with an increased risk of MACE (N = 7404, risk ratio [RR]: 0.90, 95% CI = 0.69-1.17), *I*^2^ = 0%. In addition, none of the individual MACE outcomes indicated a statistically significant finding (see Table 2 and Figure 2).

**Table 2.** Pooled Rates of MACE for Hydroxychloroquine/Chloroquine Versus Control.

Outcome	Group; no. of events, n/N		Effect estimate (95% CI)
	HCQ-CQ (N = 3777)	Control (N = 3627)	
Any MACE	125	130	RR: 0.90 (0.69-1.17)
Mortality	107	113	RR: 0.89 (0.69-1.15)
Sudden cardiac death	0	0	NA
Nonfatal cardiac arrest	10	4	RR: 1.23 (0.71-2.12)
Torsades de pointes	0	0	NA
Ventricular tachyarrhythmia	7	6	RR: 1.01 (0.58-1.74)
Seizure	3	4	RR: 0.94 (0.53-1.67)
Syncope	0	4	RR: 0.88 (0.48-1.61)

Abbreviations: CQ, chloroquine; HCQ, hydroxychloroquine; MACE, major adverse cardiac events; NA, not applicable; RR, risk ratio.

### Mortality

Hydroxychloroquine-chloroquine was not associated with an increased risk of mortality (N=7404, RR: 0.89, 95% CI 0.69- 1.15), I2 =0%.

In prespecified subgroup analyses by treatment drug (HCQ or CQ vs control), HCQ was not associated with an increased risk of mortality (N = 6677, RR: 0.99, 95% CI = 0.73-1.36) while CQ was associated with a protective effect of mortality (N = 622, RR: 0.64, 95% CI = 0.48-0.85). The remainder of the subgroup analyses, including age, sex, prespecified outcomes, cardiac exclusion criteria, and active therapy, did not reveal statistically significant findings.

### Cardiac Arrhythmias

There were no reports of torsades de pointes in our included trials. There were also no reports of sudden cardiac deaths in our included trials. One trial reported 10 nonfatal cardiac arrests in the HCQ arm, with 4 in the placebo arm.^49^ The pooled effect estimate was RR: 1.23, 95% CI = 0.71-2.12.

Two trials reported ventricular tachyarrhythmia, one reporting 5 ventricular tachyarrhythmias in the HCQ arm and 6 in placebo^49^ and another reporting 2 ventricular arrhythmias in the HCQ arm and none in the placebo arm.^40^ There were no reports of ventricular tachyarrhythmias in the CQ trials. The pooled effect estimate for ventricular tachyarrhythmia was RR: 1.01, 95% CI = 0.58-1.74.

### Seizures and Syncope

Two trials reported seizures, with a total of 3 events in HCQ-CQ compared with 4 in placebo.^49,65^ The pooled effect estimate for seizure was RR: 0.94, 95% CI = 0.53-1.67. Syncopal events were also reported in 2 trials, with no events in HCQ-CQ compared with 4 events in placebo arm.^39,64^ The pooled effect estimate for syncope was RR: 0.88, 95% CI = 0.48-1.61.

### Subgroup and Sensitivity Analyses

**Figure 3.**
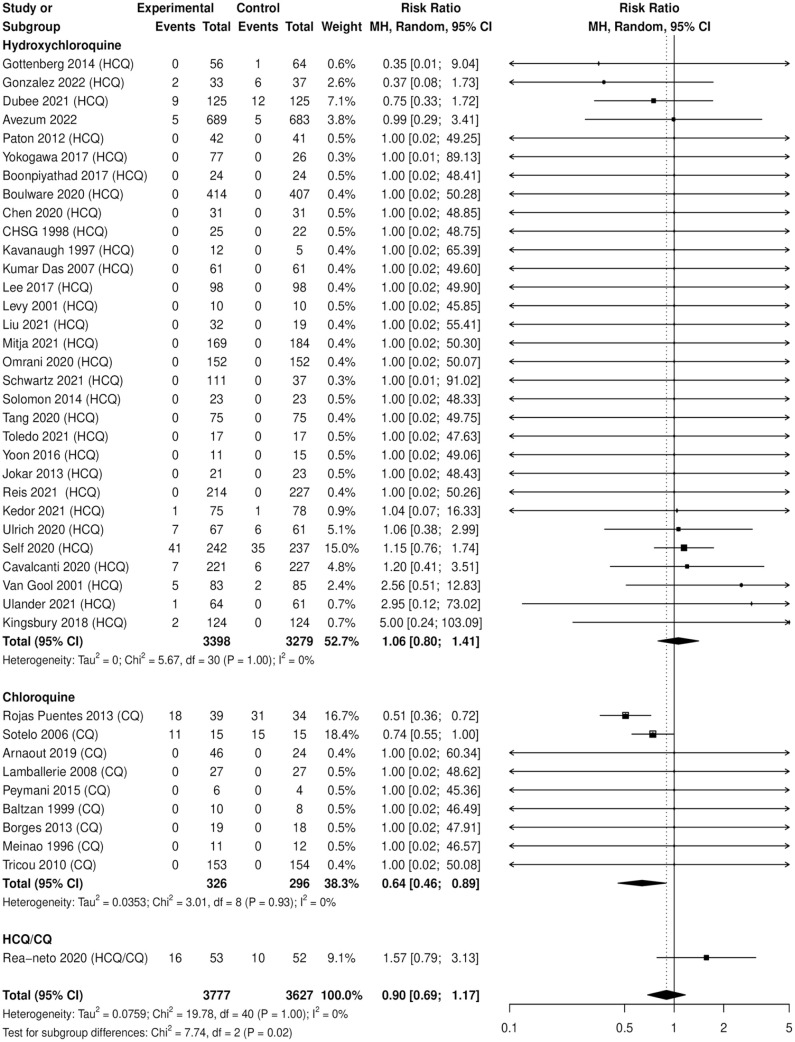
Number of individuals with ≥1 MACE, by study drug. Abbreviations: CQ, chloroquine; HCQ, hydroxychloroquine; MACE, major adverse cardiac events; MH, Mantel-Haenzel.

A summary of subgroup and analyses are available in Tables 3 and 4 and Figure 3. Chloroquine was associated with a protective effect of MACE (N = 622, RR: 0.64, 95% CI = 0.46-0.89), with the remainder of the subgroup analyses not statistically significant by age, sex, exclusion of patients with cardiac risk, active comparator, or whether QTP or a MACE was a specified outcome of interest. Using the Peto statistical method to exclude zero-zero event trials (14 RCTs, N = 3528) produced similar effect estimates than in our primary analyses (Peto odds ratio: 0.84, 95% CI = 0.48-1.44). As well, effect estimates for overall MACE remained robust when removing all-cause mortality from the analyses (RR: 0.79, 95% CI = 0.48-1.31). In the post hoc meta-regression, there was no association between cumulative base dose and MACE (*P* = 0.535).

**Table 3.** Subgroup Analyses for MACE.

Subgroup	No. of RCTs	Group; no. of events, n/N		Effect estimate (95% CI)
		HCQ-CQ	Control	
Overall	41	125/3777	130/3627	RR: 0.90 (0.69-1.17)
HCQ vs control	31	80/3398	74/3279	RR: 1.06 (0.59-1.41)
CQ vs control	9	29/326	46/296	RR: 0.64 (0.46-0.89)
Female > 50%	26	47/2716	58/2651	RR: 1.04 (0.65-1.65)
Female ≤50%	13	78/1023	72/934	RR: 1.07 (0.75-1.55)
Age ≥65 years	3	21/275	20/271	RR: 1.00 (0.55-1.82)
Age <65 years	35	104/3431	110/3305	RR: 0.88 (0.65-1.19)
QTP specified outcome	8	61/1681	52/1667	RR: 1.14 (0.81-1.60)
QTP not specified outcome	33	64/2096	78/1960	RR: 0.82 (0.59-1.14)
MACE specified outcome	13	115/2329	120/2241	RR: 0.88 (0.65-1.20)
MACE not specified outcome	28	10/1448	10/1386	RR: 1.01 (0.55-1.87)

Rea-Neto et al^67^ not included in subgroup by drug (HCQ-CQ combined). Yoon et al^57^ and De Lamballerie et al^61^ not included in subgroup on % female (missing data). Liu et al,^43^ De Lamballerie et al,^61^ and Kavanaugh et al^38^ not included in subgroup on age (missing data).

Abbreviations: CQ, chloroquine; HCQ, hydroxychloroquine; QTP, QT-prolongation; MACE, major adverse cardiac events; RR, risk ratio.

**Table 4.** Sensitivity Analyses for the Outcome of Overall MACE.

Statistical method	Statistical model	No. of RCTs	Group; no. of events, n/N		Effect estimate (95% CI)
			HCQ-CQ	Control	
(Default) TACC – 0.5	Random (MH)	41	125/3777	130/3627	RR: 0.90 (0.69-1.17)
Peto (excluding zero-zero trials)	Random OR	14	125/1830	130/1698	OR: 0.84 (0.48-1.44)
Removing all-cause mortality from MACE	Random (MH)	41	8/3777	16/3627	RR: 0.79 (0.48-1.31)

Abbreviations: CQ, chloroquine; HCQ, hydroxychloroquine; MACE, major adverse cardiac events; MH, Mantel-Haenszel; OR, odds ratio; RCTs, randomized controlled trials; RR, risk ratio; TACC, Treatment Arm Continuity Correction.

### Publication Bias

Statistical evaluation of publication bias using Harbord test on overall MACE does not suggest funnel plot asymmetry (publication bias) (*P* = 0.0622). Funnel plots are presented in Supplemental Appendix 3.

## Discussion

We believe this to be the first scoping review with meta-analysis of HCQ-CQ trials to evaluate the cardiac safety of these medications. Despite being the subject of prescribing alerts related to cardiac events, we did not find that treatment with HCQ-CQ was associated with an increased risk of mortality, ventricular tachyarrhythmias, seizures, or syncope compared with control. There were no reports of torsades de pointes or sudden cardiac death. We observed that treatment with CQ itself was associated with a 36% reduction in risk of overall MACE (RR: 0.64, 95% CI = 0.46-0.89). This finding was mostly driven by deaths. This was unexpected since CQ is generally considered to have a less favorable safety profile than HCQ. For example, of the 2 CQ trials that reported MACE, both were in cancer patient populations where CQ was used as an adjunct to proven cancer therapy (radiotherapy and chemotherapy).^64,65^ Chloroquine’s efficacy for treating cancer remains an ongoing area of research, with both preclinical and human trials ongoing investigating its use to increase the efficacy of other anticancer agents.^69^

Medications including HCQ and CQ considered as “known risk” medications on the CredibleMed’s QTdrugs list are evaluated using ADECA (Adverse Drug Event Causality Analysis) to stratify them by their ability to cause QT prolongation and/or torsades de pointes.^16^ The process uses the classic Bradford Hill criteria to assess available laboratory, adverse event databases, and “clinical evidence” for drug safety. Known risk medications are considered to have “substantial evidence” for associations with both QTP and torsades de pointes, but the evidence can be small case series or individual case reports.^16^ By modern GRADE level of evidence standards, many “known risk” medications have been so classified based on very low-quality evidence.^70^ This may be a reasonable precaution for drugs which are new to market, or which are not standard of care for diseases or conditions which themselves are known dangers to patient health. The surrogate properties of QTP are suspect for many drugs and subsequent higher quality evidence has not been incorporated into the criteria. For example, the vast majority of patients with QTP do not develop cardiac events, and torsades de pointes may occur without QTP or even with QT shortening.^71-73^ The World Health Organization itself has commented that QTP and its relationship with torsades de pointes is not straightforward.^18^

There are strengths and limitations to this review. Strengths include a focus on randomized controlled trials, forming the highest level of evidence in terms of minimizing bias including balancing differences in concomitant therapies and other potential confounders between arms. This review also excluded trials with healthy volunteers, allowing for the representation of diverse patient demographics and clinical populations. For example, various daily doses (HCQ ranged from 300 to 1200 mg, CQ ranged from 150 to 600 mg) and a variety of conditions were studied, including rheumatologic, infectious, immunologic, and oncologic conditions. In addition, we followed the recommended methods to include all available data in our meta-analyses, including zero-zero event trials.^23,74,75^ Even when zero-zero event trials were removed from the meta-analyses, the effect estimates did not significantly change, suggesting these findings are robust despite the diversity in therapeutic doses and durations.

There are limitations to our approach. Randomized trials tend to recruit healthier patients, which would tend to lower the frequency of events in both groups. Our review identified 255 major cardiac events, which is a reasonable number, but an elderly rheumatic population with co-existing cardiovascular disease would be expected to have a higher event rate. In addition, the trade-off of restricting our included studies to randomized trials in order to avoid the biases and confounding of observational studies means a loss of power to detect rare adverse events.^76^ We calculated our study to have 80% power to rule out an actual 1% MACE outcome rate in the underlying population.^77^ While a frequent criticism of RCTs is their lack of focus on harm, the usual reporting of serious adverse events would include all of our MACE outcomes. As a scoping review, another limitation is the lack of certainty of evidence (GRADE) evaluation.^70^

### Relevance to Patient Care and Clinical Practice

The American College of Rheumatology recommends HCQ between 200 and 400 mg daily, and the Centers for Disease Control and Prevention (CDC) advises up to 500 mg weekly for CQ in the context of malaria.^78,79^ As only a handful of included trials used above these recommended doses,^29,38,45,51,60,66^ and none of these trials reported any MACE, the findings of this review are reassuring for patients prescribed HCQ and CQ at usual doses. Evaluating the proarrhythmic effect of HCQ-CQ is necessary for assessments of cardiac safety, and this review found that these serious events were not more common in HCQ-CQ arms than comparator arms in the high-quality literature. Clinical decision-making and prescribing guidelines for HCQ and CQ should similarly consider factors such as the event rate, severity of outcome, and quality of evidence. Clinically, there remains a major clinical need for a valid clinical prediction rule for QT-prolonging medications such as HCQ-CQ and MACEs, as existing support is limited to surrogate outcomes.^80^ This research supports the ongoing effort to emphasize high-quality evidence and patient-important outcomes such as MACEs, over surrogates like QTP.

## Conclusions

We found no high-quality evidence that HCQ-CQ increases the risk of MACE. Further research should seek to develop a clinical prediction rule for known risk QT-prolonging medications such as HCQ-CQ and MACE so that decision support can be improved.

## Supplemental Material

sj-docx-1-aop-10.1177_10600280231204969 – Supplemental material for Hydroxychloroquine-Chloroquine, QT-Prolongation, and Major Adverse Cardiac Events: A Meta-analysis and Scoping Review Supplemental material, sj-docx-1-aop-10.1177_10600280231204969 for Hydroxychloroquine-Chloroquine, QT-Prolongation, and Major Adverse Cardiac Events: A Meta-analysis and Scoping Review by Michael Cristian Garcia, Kai La Tsang, Simran Lohit, Jiawen Deng, Tyler Schneider, Jessyca Matos Silva, Lawrence Mbuagbaw and Anne Holbrook in Annals of Pharmacotherapy
